# Chronic Hepatitis C Infection Treated with Direct-Acting Antiviral Agents and Occurrence/Recurrence of Hepatocellular Carcinoma: Does It Still Matter?

**DOI:** 10.3390/v16121899

**Published:** 2024-12-10

**Authors:** Carlo Smirne, Maria Grazia Crobu, Irene Landi, Nicole Vercellino, Daria Apostolo, David James Pinato, Federica Vincenzi, Rosalba Minisini, Stelvio Tonello, Davide D’Onghia, Antonio Ottobrelli, Silvia Martini, Christian Bracco, Luigi Maria Fenoglio, Mauro Campanini, Alessandro Maria Berton, Alessia Ciancio, Mario Pirisi

**Affiliations:** 1 Department of Translational Medicine, Università del Piemonte Orientale, 28100 Novara, Italy; nicole.vercellino@uniupo.it (N.V.); daria.apostolo@uniupo.it (D.A.); david.pinato@imperial.ac.uk (D.J.P.); federica.vincenzi24@gmail.com (F.V.); rosalba.minisini@med.uniupo.it (R.M.); stelvio.tonello@med.uniupo.it (S.T.); davide.donghia@uniupo.it (D.D.); mauro.campanini@maggioreosp.novara.it (M.C.); mario.pirisi@med.uniupo.it (M.P.); 2 Internal Medicine Unit, Maggiore della Carità Hospital, 28100 Novara, Italy; 3 Laboratory of Molecular Virology, Maggiore della Carità Hospital, 28100 Novara, Italy; mcrobu@cittadellasalute.to.it; 4 Clinical Biochemistry Laboratory, City of Health and Science University Hospital, 10126 Turin, Italy; 5 Emergency Medicine Department, Michele e Pietro Ferrero Hospital, 12060 Verduno, Italy; irene.landi30@gmail.com; 6 Department of Surgery & Cancer, Imperial College London, Hammersmith Hospital, London SW7 2AZ, UK; 7 Gastroenterology Unit, City of Health and Science University Hospital, 10126 Turin, Italy; aottobrelli@cittadellasalute.to.it (A.O.); smartini@cittadellasalute.to.it (S.M.); alessia.ciancio@unito.it (A.C.); 8 Department of Internal Medicine, Santa Croce e Carle Hospital, 12100 Cuneo, Italy; bracco.c@ospedale.cuneo.it (C.B.); fenoglio.l@ospedale.cuneo.it (L.M.F.); 9 Division of Endocrinology, Diabetes and Metabolism, City of Health and Science University Hospital, 10126 Turin, Italy; alessandromaria.berton@unito.it; 10 Department of Medical Sciences, University of Turin, 10126 Turin, Italy

**Keywords:** direct-acting antiviral agents, hepatitis C virus, hepatocellular carcinoma, interferon, liver carcinogenesis, sustained virological response, advanced fibrosis, occurrence, recurrence, cirrhosis

## Abstract

Hepatitis C virus (HCV) infection is a significant risk factor for liver cirrhosis and hepatocellular carcinoma (HCC). Traditionally, the primary prevention strategy for HCV-associated HCC has focused on removing infection through antiviral regimes. Currently, highly effective direct-acting antivirals (DAAs) offer extraordinary success across all patient categories, including cirrhotics. Despite these advancements, recent studies have reported that even after sustained virologic response (SVR), individuals with advanced liver disease/cirrhosis at the time of DAA treatment may still face risks of HCC occurrence or recurrence. Based on this premise, this review tries to shed light on the multiple mechanisms that establish a tumorigenic environment, first, during chronic HCV infection and then, after eventual viral eradication by DAAs. Furthermore, it reviews evidence reported by recent observational studies stating that the use of DAAs is not associated with an increased risk of HCC development but rather, with a significantly lower chance of liver cancer compared with DAA-untreated patients. In addition, it seeks to provide some practical guidance for clinicians, helping them to manage HCC surveillance of patients who have achieved SVR with DAAs.

## 1. Introduction

The advent of direct-acting antiviral agents (DAAs) has drastically changed the approach to hepatitis C virus (HCV) infection, a leading cause of hepatocellular carcinoma (HCC) in several countries [1]. In this respect, with interferon (IFN)-based therapies, achieving with interferon (IFN)-based therapies sustained virologic response (SVR), i.e., undetectable HCV-RNA at 12–24 weeks after completion of treatment, reduces the incidence of HCC. However, recent data have suggested an increased risk of HCC after IFN-free treatments (e.g., the aforementioned DAA regimens) [2]. One proposed mechanism to explain this trend involves a deregulation of the antitumor response following the abrupt reduction in HCV viral load, potentially promoting the progressive development of pre-existing neoplastic clones. In this respect, the lack of both good-quality prospective randomized clinical trials and adequate planned clinical goals in the early era of DAAs initially complicated the interpretation of data. Nevertheless, primary evidence suggests that DAAs do not have a carcinogenic effect per se. Instead, they may lead to the early occurrence of previously existent neoplastic liver diseases that were underestimated at the time of antiviral treatment [3]. Therefore, patients with HCV infection should be encouraged to initiate DAA therapy to prevent cirrhosis and subsequent HCC. However, extensive screening to exclude HCC is recommended before drug administration, especially in patients with cirrhosis or other significant hepatic comorbidities. Also, since viral eradication does not entirely eliminate the possibility of ongoing liver disease and HCC, lifelong monitoring is advised for high-risk patients, even after achieving SVR [4,5].

**Figure 1:**
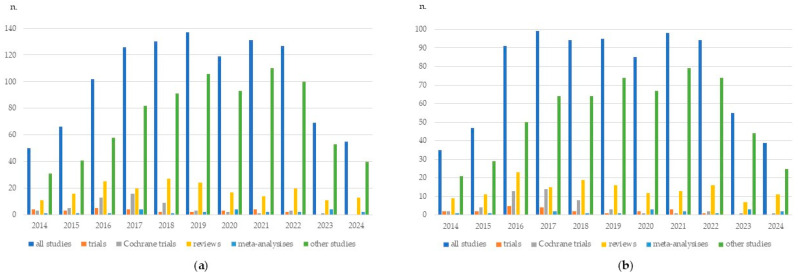
Available publications (updated to 1 October 2024) concerning the risk of hepatocellular carcinoma development after hepatitis C virus eradication with direct-acting antivirals. Trials include both clinical trials and randomized controlled trials; Cochrane Central Register of Controlled Trials is considered separately, after checking for possible overlaps with PubMed/MEDLINE databases. Reviews include both systematic and non-systematic reviews. The year 2024 refers to the literature published from 1 January 2024 to 30 September 2024. (**a**) Literature analysis including all stages of liver fibrosis; (**b**) literature analysis including only cirrhotic patients at the time of antiviral treatments.

The present review aims to report the most up-to-date evidence on the epidemiology and mechanisms of hepatocarcinogenesis following DAA-induced HCV eradication. In addition, we present an analysis of the now well-established risk factors for the development of HCC in patients with active HCV infection, highlighting both shared and distinct mechanisms underlying the two conditions. The complexity of this issue, which has prompted extensive scientific debate, is underscored by the huge amount of evidence produced in recent years. However, as previously stated, the quality of the research is often variable, and conflicting results are common. As an indirect confirmation of this, although a systematic review is beyond the scope of this research, we report a purely numerical count (updated to October 2024) of all relevant peer-reviewed publications in English within the literature on this topic (Figure 1). Studies were identified by searching the PubMed, MEDLINE, Scopus, Web of Science, and Cochrane Library databases, as detailed in Supplementary Table S1. However, the majority of these studies lacked high levels of evidence. Indeed, only a few clinical trials were identified, and randomized controlled trials were even rarer. To date, most evidence derives from expert opinions, but scoping or integrative reviews far exceed systematic reviews. The vast majority of the available documents (74.8% of the total) focused on cirrhosis, notoriously being the patient subgroup most prone to HCC development.

## 2. Chronic Hepatitis C: Clinical Management and Debated Issues

### 2.1. Clinical Challenges in HCV Management

Globally, around 50.7 million people have chronic HCV infection, with a reported prevalence of 0.6% and an annual incidence of 1.04 million [6]. Indeed, the real prevalence is likely to be underestimated since approximately 80% of infections remain non-diagnosed worldwide [7,8,9,10,11,12,13,14].

Interestingly, HCV infection was one of the leading causes of HCC until the early 2000s, at least in the Western world. This trend seems to be diminishing, while other etiologies are emerging. These include alcoholic (ALD) and non-alcoholic (NAFLD) liver diseases, the latter currently referred to as metabolic dysfunction-associated steatotic liver disease (MASLD) and the resulting mixed disorder consisting of both MASLD and increased alcohol intake (MetALD) [15,16,17,18,19].

In any case, the introduction of safe and effective DAAs in clinical practice has now made the elimination of this public health burden achievable in the medium term, as stated in the World Health Organization (WHO) global hepatitis strategy [20]. Moreover, prospective studies on DAA-based therapies have demonstrated the benefit of HCV clearance both in liver- and non-liver-related mortality [21,22,23,24,25]. More details on some important hepatitis C-related clinical issues are provided in the Supplementary File S1 [7,26,27,28,29,30,31,32,33,34,35,36,37,38,39,40,41,42,43,44,45,46,47,48,49,50,51,52,53,54,55,56,57,58,59,60,61,62,63,64,65,66].

### 2.2. Follow-Up of Patients Who Achieved Virologic Response

Handling of patients who have achieved SVR for hepatitis C infection after DAA treatment remains a subject of current considerable interest. According to the American Gastroenterological Association (AGA), subjects with less severe fibrosis (i.e., F0-F2 METAVIR stages) [67] do not require continued follow-up, particularly in relation to ultrasound monitoring for HCC screening. In fact, due to recognized lack of progression, non-cirrhotic subjects who achieve SVR should receive the same medical assistance as those who have never been infected with HCV, unless they remain at risk for non-HCV-related liver disease such as MASLD or ALD [68].

The approach is necessarily different in individuals who have been cured of the infection but have already reached the stage of pre-cirrhosis (METAVIR F3) or cirrhosis (METAVIR F4). In this subset of patients, decompensated liver disease rarely occurs during follow-up, and overall survival is thus prolonged, compared with patients who have not achieved virologic response. Also, bleeding from esophageal varices is uncommon [28,69]. Nevertheless, these subjects remain at risk of HCC development (whether recurrent or de novo) even after SVR has been achieved. Indeed, this remains a partially unresolved issue, although it was established that irrespective of the specific antiviral treatment, the risk persists for an unspecified duration (most authors agree that it is at least 10 years) even after virologic response. Therefore, it is now widely established that all these patients should undergo HCC surveillance regardless of viral eradication, i.e., an abdominal ultrasound every six months (with or without α-fetoprotein (AFP) testing), despite any fibrosis regression [70,71,72,73]. Instead, for years, researchers have debated whether there is a possible link between the specific use of DAA agents and the insurgence of HCC. In any case, as discussed further in this article, numerous publications have suggested that if such risk exists, it is very low. The reported increase in HCC rates after DAA treatment is likely to be due to the fact that the first cohorts of patients treated were preselected to be at higher risk for HCC.

## 3. Incidence and Mechanisms of HCC Occurrence in HCV Patients

### 3.1. Epidemiology of HCC

In light of the variable prevalence of underlying risk factors, the global incidence of HCC is heterogeneous. Over 70% of cases occur in Asia, with half of them in China alone, 10% in Europe, 7.8% in Africa, 5.1% in North America, 4.6% in Latin America, and 0.5% in Oceania [74]. In 2015, there were an estimated 854,000 new primary liver cancer cases and 810,000 cancer-related deaths worldwide [75]; about 75–85% of these liver tumors were represented by HCC, making it a major health problem [76].

HCC mainly occurs in chronic liver disease and cirrhosis, accounting for 80–90% of total cases. The incidence rate for HCC in patients with cirrhosis from any cause is about 2.3 (95% confidence interval (CI): 2.2–2.4) per 100 person-years (py) [77]. However, aside from the stage of liver disease, HCC is a highly complex condition with multiple determinants implicated in its etiology. The primary known risk factors include hepatitis B virus (HBV) and HCV, ALD, and MASLD (including diabetes mellitus (DM) and obesity) [78]. Overall, HBV and HCV are responsible for about 60–85% of all cases of HCC, as both these chronic infections can result in the development of cirrhosis, while 11% are due to alcohol, and about 10% are attributable to other causes [79]. Generally speaking, HBV is the main factor that leads to HCC in most countries in Asia and Africa. The risk can be implemented by the exposure to region-specific hepatotoxins, such as the food contaminant aflatoxin B1, which is endemic in many hot climates, or aristocholic acid, commonly used in Chinese herbal medicine, which may act as a cofactor in liver carcinogenesis. Instead, HCV is a prevailing cause in most European and North American countries, as well as in Japan [80].

Nonetheless, the current epidemiologic landscape of HCC is predicted to change due to the rising occurrence of non-viral cirrhosis, at least in the Western world. In the future, the early onset of MASLD and ALD among younger individuals could attenuate or even surpass the well-known improvements in HCC incidence that are attributable to the managing of HBV and HCV infections. Indeed, it is estimated that during the next two decades, non-alcoholic steatohepatitis (NASH), which is also named metabolic dysfunction-associated steatohepatitis (MASH) and is the evolving form of MASLD, will be the most common cause of cirrhosis and thus, of HCC in the more industrialized nations [81]. For instance, in the USA, up to 20–30% of individuals already have MASLD or some features of metabolic syndrome, in addition to 25% with ALD [82]. In Europe, while obesity is increasing, it still accounts for a smaller proportion of HCC cases (around 16%) compared with the USA [83].

The previous considerations are important because it should be noted that a minor yet significant proportion of HCC cases (12–20%) develop on non-cirrhotic liver. Patients with MASLD account for the majority of HCC cases without underlying advanced fibrosis, with around 30% of such liver cancers occurring in this population [84]. However, traditional risk factors (such as male sex, older age, Hispanic origin and DM, as well as hepatic cirrhosis), remain the most critical risk factors for HCC in this setting [81].

### 3.2. Occurrence of HCC During Chronic Hepatitis C

HCV was recognized as an independent risk factor for HCC, particularly in cirrhotic subjects, shortly after its discovery. Indeed, this virus is widely acknowledged as a major cause of both cirrhosis and HCC, as discussed previously [77]. This understanding began with case reports of HCC arising during chronic non-A, non-B hepatitis [85], and with the observation from Japan of an increasing incidence of non-HBV-related HCC [86]. Since HCV was identified as the principal causative agent of blood-borne non-A, non-B hepatitis, researchers have been able to study more closely the link between this chronic viral infection and HCC. Although HCV infection prevalence is highly variable among patients with HCC, sometimes even within the same geographical area, it appears to be a significant contributor to HCC. As a matter of fact, most patients with HCC who test positive for anti-HCV antibodies also have detectable HCV RNA in serum, hepatocytes, and even in tumor tissue, confirming that an active infection is ongoing [87,88,89].

The exact mechanisms by which hepatitis C leads to HCC are not fully understood. The next section discusses the most up-to-date evidence in the field of etiopathogenesis. Regardless of the direct possible viral mechanisms involved, whether alone or in combination, most data on HCV have centered on its indirect pro-tumoral role, it being a known causative agent for cirrhosis. Indeed, most patients with HCV-related HCC also have cirrhosis or at least, advanced hepatic fibrosis [90]. On the other hand, cirrhosis is an essential precursor of hepatic malignancy, regardless of its etiology, and at autopsy, as many as 10% to 15% of patients are found also to have HCC [91]. Yet, not all cirrhosis cases have the same risk of developing hepatocarcinoma. In this respect, HCV-related cirrhosis appears to be more cancer-prone than the forms caused by many other etiologies, supporting the idea that HCV may have direct carcinogenic effects [92].

Although the association between HCV infection and subsequent HCC is well known, determining HCC risk for the individual patient remains challenging. This difficulty is due to the overall natural history of HCV infection, which is quite variable, and to the long-term risks that are complex to quantify because of the prolonged duration involved. Nevertheless, the risk of developing HCC in HCV-positive subjects can be extrapolated from the results of HCV natural history studies and reports on HCC development in individuals with HCV-related cirrhosis. Assuming that 20% of patients develop cirrhosis within ten years from infection, the risk of HCC thereafter is estimated to be 1–4% per year, which would mean that from 9.6% to 33.5% of HCV cirrhotic subjects develop cancer after 20 years. However, these estimates must be interpreted with caution, because most studies have included very heterogeneous patient populations. Furthermore, the additional risk factors of age and sex as well as cofactors like alcohol use and other environmental exposures should be used to adjust these estimates [93].

The considerations mentioned above should not overshadow the fact that while HCC predominantly arises in the context of cirrhosis, nearly 15% of HCV patients who develop HCC have no definite cirrhosis [14]. This supports the hypothesis that HCV can cause HCC directly, similarly to MASLD and HBV infection, although to a lesser extent [84].

### 3.3. HCC Pathogenesis in Chronic HCV Infection

The development of HCC from HCV infection, as detailed in the following paragraphs, can be due, alternatively or synergistically, to the following factors: (a) direct virus-induced cellular programming; (b) indirect host-related inflammatory response; (c) overlapping host metabolic bystander effect [14].

#### 3.3.1. Direct Viral Oncogenic Mechanisms

Hepatocytes adapt to chronic HCV infection mainly through changes in the programming of their cell survival, which can be induced by various structural and/or non-structural viral proteins altering different intracellular cascades. A pivotal factor in this adaptation is the stress response of the hepatocytic endoplasmic reticulum (ER). This, in turn, can be caused by virus-induced cellular protein modifications, occurring at the synthesis stage or any of the post translational phases (such as degradation or folding). ER stress can then trigger signals that initiate cell disruption and inflammation; if the infection persists, this can lead to progressive hepatic damage and fibrosis. Then, when the cirrhosis stage is reached, this exaggerated stress ultimately favors HCC occurrence, basically provoking a switch to unregulated cell proliferation.

Direct virus-induced cellular reprogramming includes the down-regulation of tumor suppressor genes and the promotion of genomic instability consisting of an increased rate of chromosome gains and losses. In more detail, the multiple intracellular cascades that are most commonly affected during HCV-mediated hepatocarcinogenesis can induce alterations in the setting of cell proliferation, since dysregulation in cellular replication or cell-cycle control are important features of HCC development [94,95,96,97,98]. Additional alterations include uncontrolled angiogenesis (being the generation of new vessels crucial for all cancers) [99] and epigenetic modifications (such as DNA methylation, histone modifications and the production of non-coding RNAs, which in turn can all cause changes in gene expression and, ultimately, tumorigenesis) (Table 1) [100,101,102,103,104,105,106,107].

#### 3.3.2. Indirect Host-Related Inflammatory Response Mechanisms

Chronic HCV replication disrupts liver immune tolerance, triggering protracted inflammation that can result in hepatic fibrosis, cirrhosis, and HCC. Moreover, several HCV proteins are also able to compromise the cytotoxic and regulatory activities of immune cells, such as antigen-presenting cells (APCs), natural killer (NK) cells, or CD4 and CD8 T cells, eliciting aberrant inflammatory cytokine production from the innate immune response and bypassing the hosts’ antiviral adaptive immunity, ultimately resulting in further progression of hepatic damage (Table 2A) [108,109,110,111].

#### 3.3.3. Bystander Oncogenic Mechanisms

Possible concomitant non-viral liver comorbidities can accelerate HCV-mediated fibrogenesis, cirrhosis development, and HCC. Among these, metabolic disorders such as obesity and DM have a pivotal role, but alcohol assumption and co-infections, e.g., with HBV or human immunodeficiency virus (HIV) also have a definite role. This evidence replaces or at least complements the previous paradigm, which was mainly tumor-centered (based on the classical activation of oncogenes and/or loss of tumor suppressors), and may be a critical point that finally starts to explain why HCC can still develop in the absence of HCV after DAA virological response, as detailed in the next section (Table 2B) [112,115,116,117,118,119,120,121].

For instance, DM increases the risk of HCC development in HCV-infected patients, particularly in patients with early DM diagnosis and concomitant liver cirrhosis [113]. However, further studies with longer follow-ups are required to investigate the effective influence of DM on survival rate and to determine the potential benefits of intensified HCC screening in diabetic cirrhotics [114].

The mechanisms of HCC development in all these cases are expected to reside primarily in various forms of mainly sterile bystander inflammation involving neighboring cells, especially when the cirrhosis stage is finally reached. Cirrhotic microenvironment may promote HCC mainly through contact-dependent cell–cell mechanisms. So, when adhesion complexes are variously altered during cirrhosis, normal tissue homeostasis is disrupted and adverse consequences can occur. These include altered apoptosis and pyroptosis of non-transformed cells to enhanced expansion/self-renewal or increased susceptibility of cancer stem cells to chemical hepatocarcinogenesis. Crucial to these processes is the abnormal expression of Cx26 and Cx32 proteins, which are key elements of cellular gap junctions [122].

### 3.4. Occurrence of HCC After Hepatitis C Virus Eradication

HCV eradication represents a milestone of HCC prevention, as it both reduces chronic inflammation and prevents the previously cited direct oncogenic viral mechanisms. Viral clearance is also associated with a decrease in portal venous pressure, resulting in reduced mortality, at least for subjects with overt cirrhosis [123]. As expected, HCC risk reduction is significant upon achieving SVR rather than the virus merely being suppressed temporarily [124]. This is true irrespective of the baseline fibrosis stage at which the patient is treated, but it is obviously more evident in individuals with more severe liver disease (i.e., pre-cirrhosis and overt cirrhosis) [69,82]. Nonetheless, SVR definitely still accounts for some substantial persistent risk of HCC, as described in more detail below [125].

#### 3.4.1. Occurrence of HCC After SVR Achieved with (PEG)-Interferon

Historically, IFN-based therapies, i.e., standard IFN-α or pegylated (PEG)-IFN-α in combination with ribavirin (RBV), were the standard of care for hepatitis C for several years, as described in Supplementary File S1. These treatments, however, were recognized as bearing substantial residual risk of hepatocarcinogenesis even after SVR. Not being the focus of the present review, all these aspects are extensively detailed in Supplementary File S2 [21,63,69,126,127,128,129,130,131,132,133,134,135,136,137,138,139,140,141,142,143,144,145,146,147,148,149,150,151,152].

#### 3.4.2. Occurrence of HCC After SVR Achieved with DAAs

HCV clearance, as previously reported, should avoid or at least reduce the majority of severe complications of chronic hepatitis C, including HCC occurrence. As the latter is mainly related to HCV’s direct (i.e., oncogenic) and indirect effects (such as the emergence of possible cirrhosis with necro-inflammatory activity and the failure of immune surveillance due to escape mechanisms), it is plausible that viral clearance after DAAs reduces HCC development by reversing both mechanisms. However, based on the assumption that DAA-induced SVR does not completely eradicate the HCC risk, in analogy to what we previously reported for IFN-containing regimens, its exact role in the long-term incidence or recurrence of HCC remains a matter of debate [125,153,154,155].

According to AGA recommendations, DAA treatments resulted in nearly 70% reduction in HCC risk for SVR patients. This effect was evident much earlier than in IFN-based regimens (within 3–6 months), and it still increased over time. The absolute yearly risk of HCC was around 0.90% in the largest cohorts with virological response, even after accounting for socio-demographic and clinical differences among participants. In contrast, most studies agreed that non-SVR subjects remained at substantial risk for liver cancer. Summarizing similar findings to those reported with IFN-containing regimens, the incidence of HCC was highest in patients with cirrhosis and no SVR (3 per 100 py), followed by cirrhosis and SVR (2 py), no cirrhosis and no SVR (1 py), and no cirrhosis and SVR (around 0.2 py). Most of these multivariable models confirmed that achieving SVR was associated with a significant reduction in HCC risk, regardless of the specific DAA used. Moreover, most studies agreed that among treated persons cancer risk was not higher in those receiving DAAs (DAAs only or DAAs + PEG-IFN) compared with those treated with PEG-IFN only (hazard ratio (HR): 1.1) [156,157,158,159,160,161,162,163,164,165,166,167,168,169]. This effect was similar across different races and ethnic groups [170,171,172].

In essence, cirrhotic patients at the start of DAA regimens carry greater risk of HCC occurrence and recurrence after viral eradication. Although most studies confirmed a significant reduction in this risk for such patients, a few reports, especially between 2015 and 2019, suggested an unexpected increase in early HCC cases. However, most recent studies have not consistently confirmed these data, and there is no present evidence to support the suggestion that DAAs may directly promote HCC [164,173]. Thus, at least as far as the risk of HCC after HCV clearance is concerned, this remains an important and partially unresolved issue when evaluating the long-term risks (very few) and benefits (many) of current antiviral treatments. These considerations are especially relevant given that current evidence on DAAs does not yet include data on adequately long follow-up after eradication [14].

So, understandably, the development of HCC after DAA-induced virological response has created a broad scientific debate which has not yet ended. The two dominant hypotheses that could explain this event are a possible carcinogenic effect of DAAs or the existence of small HCC nodules not detected by ultrasound screening before the start of treatment [125]. However, while a direct role for DAAs in hepatocarcinogenesis has never been demonstrated, further research is necessary to dissect host-related mechanisms that could determine the risk of HCC in the absence of HCV. In more detail, after fast HCV clearance achieved with DAAs, the exact contribution of a putative reduction in immune surveillance and a change of cytokine patterns remains largely unknown. It is believed that the anticancer functions of the immune system could be altered, resulting in a drastic fall of HCV-stimulated immune surveillance, possibly promoting early carcinogenesis. Moreover, HCV is known to induce genetic and epigenetic alterations such as modifications of the histone tail and DNA methylation, which are known risk factors for liver cancer and may persist long after virological response (further details are reported in Section 4.2) [14,125,174].

In any case, the multistage process of tumor development, in which HCV is involved, is usually preceded by the onset of cirrhosis (or, to a lesser extent, advanced stage F3 fibrosis), which plays a pivotal role in tumor initiation. Indeed, as described above, most cases of HCC that develop after viral eradication occur in the context of cirrhotic livers. Studies consistently showed that the absolute HCC risk remained high in the patients who had already reached the cirrhosis stage at the time of achieving SVR with DAAs (yearly risk: 1.8–2.5%) [175]. This strong evidence supports the hypothesis that the relatively high incidence of HCC in the first year after virological cure might be at least in part related to “missed” HCC cases due to unproper surveillance among at-risk patients. This scenario was particularly common in the early years following the introduction of DAAs, when many patients had advanced disease or were unable to receive IFN therapy due to intolerance or contraindications. Moreover, DAA-treated cirrhotic subjects have even higher HCC risk if other risk factors are present, such as high liver stiffness, elevated AFP values, DM, or male sex, in analogy with what was described for IFN-based therapies [125,156]. To expand on the latter concept, a more extensive review of the established evidence on the similarities and differences in HCC occurrence after treatments with IFN compared with DAAs can be found in Supplementary File S3 [2,123,125,151,176,177,178,179,180,181].

## 4. Potential Mechanisms Underlying the Persistent Risk of HCC in Patients with SVR Achieved with DAAs

### 4.1. The Role of the Immunosurveillance

The reasons why HCC develops in patients who have achieved SVR following DAA treatment are controversial. The leading hypothesis is the reduction of immunosurveillance in response to the rapid decrease in viral load (Table 3A) [5].

An example of this process is the reduction in expression of endogenous IFNs. Typically, after the establishment of HCV infection, it is known that type I and type III IFNs are produced by host hepatocytes [198]. Consequently, after binding to their respective receptors, they initiate a signaling cascade through the Janus kinase (JAK)–signal transducer and activator of transcription 1 (STAT1) pathway. The downstream cellular actions are then mediated by the induction of interferon-stimulated genes (ISGs), which have antiviral and immunoregulatory effects [198,199]. DAA treatment has been proven to inhibit these processes, thereby leading to a decrease in endogenous IFN levels, both in liver and in blood [182,200]. The attenuated induction of ISGs can restore IFN-α responsiveness, contributing to DAA-induced alleviation of extrahepatic manifestations. However, these mechanisms may also taper important IFN-mediated immunomodulatory and antiproliferative actions [183,201].With particular reference to type I IFNs, these properties are quite crucial not only in the host defense against pathogens (including HCV infection itself) but also for immune surveillance against tumors [202]. Meanwhile, it must be stated that the anticancer activity of INFs is multifaceted and relies on both direct and indirect mechanisms [184].

Moreover, the eradication of HCV with DAAs can disrupt the dynamic balance of pro- and anti-inflammatory signals, thus modifying host tumor surveillance and the subject’s predisposition to HCC recurrence/occurrence [185]. The risk is particularly pronounced for the most susceptible patients, including those with severe fibrosis and splanchnic collateralization, leading to abnormal activation of liver neo-angiogenetic pathways [186].

Other studies have evaluated the possible role of NK lymphocytes in preventing the development of HCC [203,204]. For instance, after DAA treatment, a rapid decrease in the quantity of natural killer group 2 member D (NKG2D) cells, one of the most widely studied immunoreceptors, was observed; this generally correlates with early HCC occurrence. Interestingly, this decrease was not observed in patients treated with IFN-combined regimens [188,189]. However, it is worth noting that other studies have suggested at least transient functional recovery of NK cells after various DAA treatments [205].

### 4.2. The Role of HCV-Induced Epigenetic Regulations

HCV induces epigenetic alterations such as histone tail modifications and DNA methylation, as previously described in Section 3.3.1. Unlike genetic changes, epigenetic modifications are reversible, regulating gene activity and the consequent production of proteins. Few epigenetic changes persist after a DAA cure (the so-called epigenetic signature), but not after IFN treatment, possibly explaining why HCC may be more frequent in the first group, at least according to some studies (Table 3B) [190].

A paper by Hamdane et al. targeting patients with HCV infection unraveled the specific genome-wide changes in histone H3K27ac. These were associated with complex alterations in mRNAs and protein expression, which largely persisted even after SVR was obtained, with both DAAs and IFN-based therapies, especially in patients with advanced fibrosis/cirrhosis. In more detail, H3K27ac modifications were positively associated with the expression of certain oncogenes, the most relevant being sphingosine kinase 1 (SPHK1) and sex-determining region Y-box transcription factor 9 (SOX9) [191,192,193].

Perez et al. described a panel of genes that remained persistently altered in hepatocytes during HCV infection, all of which were modulated by other epigenetic markers like H3K9Ac. Their high expression correlated with HCC development and more importantly, these changes also persisted in DAA-treated patients. The authors also demonstrated that epigenetic inhibitors could revert the epigenetic signatures induced by HCV. In fact, drugs such as C646 (a specific inhibitor of H3K9Ac) or erlotinib (an inhibitor of the epidermal growth factor receptor) can restore these epigenetic alterations, thus preventing oncogenesis. The authors therefore suggested that this sort of epigenetic genome “scarring” may be a novel mechanism of HCC tumorigenesis after HCV eradication by DAAs [190].

Another example of possible HCV-induced alterations is the epigenetic silencing of the promoter of dickkopf WNT signaling pathway inhibitor 3 (DKK3) protein through aberrant hypermethylation, with consequent activation of the Wnt/β-catenin signaling pathway. A possible effector of these mechanisms is the HCV core protein, a well-recognized promoter of hepatic cancer cell growth, migration, and invasion. Therefore, DKK3 may also be a potential new diagnostic and therapeutic target for HCC, especially in presence of cirrhosis [194,195,206]. It must be said that limited data are available regarding the impact of DAA treatments on these alterations, though there is preliminary evidence suggesting that these drugs cannot restore Wnt/β-catenin signaling, even after HCV eradication [196].

To summarize, understanding epigenetic modifications is crucial for identifying both patients at significant risk of developing HCC and possible new pharmaceutical targets for the prevention of this tumor [197].

## 5. How to Estimate HCC Risk After SVR

### 5.1. When Surveillance for HCC Is Required

As previously stated, eradicating HCV through antiviral treatment does not eliminate the risk of HCC. The key question is how we can reliably estimate this risk and its changes over time, to determine whether the patient might benefit from HCC surveillance.

Patients with advanced fibrosis (F3) or established cirrhosis (F4) at the time of SVR have a recognized significant residual risk of HCC that persists after viral eradication. However, the former have lower individual risk than the latter. While there is universal agreement that both these categories of patients should undergo regular HCC surveillance, proper staging of F3 fibrosis subjects is more difficult when using only non-invasive diagnostic tests, and this may complicate HCC vigilance policies. Currently, all patients in the above-mentioned categories continue this follow-up “indefinitely” or at least, until they are eligible for potentially curative HCC therapies, because they appear to be at higher risk of HCC even years after SVR [207]. This recommendation is based on many lines of evidence. First, HCV-related pre-neoplastic genetic and epigenetic changes, as well as pre-existent monoclonal micronodules, may persist for an indefinite period after SVR, predisposing individuals to the development of HCC even long after virological cure [207]. Secondly, while fibrosis is generally expected to improve significantly following DAA treatment, as a consequence of depriving the liver of the pro-inflammatory viral trigger, it may also persist long after HCV eradication. Fibrosis could even worsen due to major liver comorbidities (such as ALD, MASLD, or HBV/HIV coinfections) and non-liver-related conditions (such as DM, obesity, or metabolic syndrome). All these processes can result in a residual, not exactly definable, persistent risk of HCC [208]. In any case, there is no universal agreement as to whether liver fibrosis regression may be a factor associated with reduced risk of HCC. As a matter of fact, while various studies have suggested that this process can actually lower the rate of HCC [164,165,167,209,210], other authors have reported that HCC occurs despite any histological stage improvement [208,211,212]. Furthermore, the proper interpretation of non-invasive methods for the assessment of liver fibrosis (namely, liver stiffness) in patients following HCV cure remains unclear, possibly complicating HCC surveillance strategies [213].

Instead, for all non-advanced fibrosis subjects who achieve SVR with DAAs, the critical question is how to reasonably estimate the residual risk of HCC and how this risk changes overtime, to determine whether the individual patient could benefit from liver cancer surveillance (so-called cost-effectiveness) [207].

### 5.2. Current Recommendations About HCC Surveillance Before and After DAAs Treatment

The main clinical recommendations for managing patients undergoing HCV therapy with DAAs can be differentiated based on HCC absence (Supplementary Table S2) or presence (Supplementary Table S3) at baseline. According to the risk factors extensively described and, above all, the stage of liver fibrosis, they also include the most important recommendations for HCC surveillance once SVR has eventually been obtained. These guidelines are based on the currently available published evidence, including observational studies and systematic reviews, and incorporate expert opinion where applicable. With minor differences, they are consistent among the leading scientific hepatological associations (AGA; American Association for the Study of Liver Diseases, AASLD; European Association for the Study of the Liver, EASL; Asian Pacific Association for the Study of the Liver, APASL) [28,156,207,214,215,216,217]. One additional consideration to bear in mind is that DAAs also influence the prognosis and management of HCC, when present, and not just the other way around [154,218,219].

It should be noted that the aforementioned recommendations on HCC surveillance apply to HCV mono-infected patients when starting antiviral treatments. Thus, although HCV patients co-infected with HBV or HIV present faster progression to liver fibrosis, cirrhosis, and/or HCC, probably due to the increased severity of liver disease, specifically increased inflammation, and although there may also be some possible carcinogenic synergy between the different viruses, to the best of our knowledge, there are no dedicated guidelines for HCC monitoring in these subjects after HCV clearance. Therefore, it is reasonable to consider these individuals as carriers of important and persistent cofactors of liver damage. For this subgroup of patients, surveillance should continue indefinitely, at least as often as in any HCV mono-infected subject bearing important comorbidities, with special concern for those with already established fibrosis [220,221,222,223,224,225].

### 5.3. Current and Future Strategies for HCC Risk Estimation

Currently, the main approach for estimating HCC risk and guiding decisions about HCC surveillance in HCV patients undergoing DAA treatment is indirect and related to the fibrosis stage, as previously reported. For instance, liver stiffness obtained by transient elastography has a good correlation with the baseline fibrosis stage, although, as mentioned above, this becomes quite unreliable after SVR is achieved [226,227]. Multiple studies have shown that it is independently associated with HCC risk, both in patients with established cirrhosis and in those at a pre-cirrhotic stage [228]. This direct correlation has also recently been proven to remain generally valid after HCV eradication with DAAs, particularly in subjects with advanced fibrosis or cirrhosis [229,230,231,232,233]. Also, shear-wave elastography (SWE) and acoustic radiation force impulse elastography (ARFI), which are novel techniques used for evaluation of liver fibrosis, have demonstrated good clinical performance in predicting HCC risk in DAA-treated subjects [229,234,235].

Reasonably, a better and more accurate approach would be to estimate HCC risk directly. Promising strategies in such a context could include simplified scoring systems, multivariable HCC risk calculators, or deep learning HCC prediction models. These tools could enable risk stratification and individualized, risk-based surveillance strategies (so-called “precision HCC screening”) in the coming years. In the future, more patient-specific genetic, epigenetic, transcriptomic, or molecular profiling may identify individual patients at particularly high risk of HCC development [207,236].

#### 5.3.1. Simplified Scoring Systems

The fibrosis-4 (FIB-4) score is a simplified method for estimating HCC risk following SVR. The scoring formula includes aspartate transaminase (AST), alanine aminotransferase (ALT), age, and platelet count, and it was originally created as a non-invasive fibrosis biomarker panel. A high FIB-4 score of 3.25 was subsequently identified as a powerful predictor of HCC risk in patients both with and without cirrhosis (Supplementary Table S4) [227,237,238]. In addition, dynamic changes in the FIB-4 score following SVR could further improve the accuracy of HCC risk prediction [207,239].

#### 5.3.2. Multivariable HCC Risk Calculators

Multivariable HCC risk calculators estimate HCC risk in chronic liver disease or cirrhotic patients, including HCV-infected subjects who have completed antiviral therapy (Supplementary Table S4).

The Veterans Health Administration (VA) generated a first prediction tool that included twelve routinely available variables (treatment response, age, gender, body mass index, ethnicity, HCV genotype, platelet count, AST, ALT, albumin, international normalized ratio, and hemoglobin) to estimate the 3-year HCC risk after antiviral treatment through a multivariable Cox proportional hazards model, in patients both with and without cirrhosis [240].

The age–male–albumin–bilirubin–platelets (aMAP) risk scoring system was recently created to assess HCC risk in chronic hepatitis patients, including HCV carriers with SVR, again both in the presence or absence of cirrhosis. This model has performed remarkably well so far but needs further validation [241].

The GALAD score is a serum biomarker-based model that predicts the presence of HCC in chronic liver disease, including HCV infection. This is a promising novel artificial intelligence algorithm derived from demographic parameters including gender and age and biochemical AFP, AFP-L3 (the L3 isoform of AFP), and des-gamma-carboxy-prothrombin (DCP) levels, without imaging. It has demonstrated a sensitivity of at least 75% and a specificity of 92% for HCC diagnosis. This score clearly outperformed methods based on the clinical significance of single biomarkers (such as AFP, AFP-L3, or DCP used separately) for early HCC detection in a large cohort of white patients with chronic hepatitis B or C. Moreover, GALAD proved to have good accuracy in identifying HCC patients, regardless of tumor burden, extent of concomitant liver disease, or baseline viral load [242]. In any case, its diagnostic performance in HCV patients should hopefully be validated in upcoming large international multicenter prospective studies. Additionally, direct comparison with ultrasound examinations should also be performed. Even more relevant, there is no current study specifically analyzing this algorithm in HCC surveillance programs after DAA therapy [242,243,244,245].

Other multivariable validated clinical models to predict HCC risk after SVR may include GES (i.e., General Evaluation Score), both as a simple score and dynamic algorithm, ADRESS (i.e., age, DM, race, etiology of cirrhosis, sex, and severity of liver dysfunction), and Watanabe [246,247,248,249,250,251], as recently demonstrated in a large comparative study [252].

#### 5.3.3. Deep Learning HCC Prediction Models

Deep machine learning models, like neural networks or complex tree-based models, are generally designed to outperform conventional linear models in prediction accuracy. Although this generally happens at the expense of interpretability or at risk of substantial overfitting, incorporating deep learning prediction and diagnostic algorithms into clinical management is presently an area of great growth, and many such tools are likely to become available in routine practice in the near future. As an example, a deep learning recurrent neural network (RNN) model predicting HCC in HCV carriers was recently released (currently limited to cirrhotic subjects), again derived from the VA (Supplementary Table S4) [253]. However, validation in the context of a virological cure is still awaited [207].

## 6. Discussion

The landscape of HCV therapy has dramatically changed over the years. The introduction of DAA therapy in fact has raised the SVR rate up to 96%, with significantly better tolerance and effectiveness compared with IFN-based regimens.

Nonetheless, despite this outstanding efficacy, many reports, mostly from retrospective studies, initially suggested a possible increased risk of HCC occurrence and/or recurrence after DAA treatment. This prompted the debate on the safety profile of these regimens and their possible association with HCC development. Many objections were soon raised concerning the unavailability of control groups, generally limited sample sizes, and short follow-up periods. After years of intense research, there are now several good-quality studies that definitively indicate no significant increase in HCC occurrence in patients who achieved sustained eradication of HCV with both IFN or DAAs. What is more, several reports have highlighted the efficacy of DAAs in substantially lowering the risk of HCC in such subjects, compared with those with either treatment failure or no therapy at all [254]. This is presumed to be a drug class effect, affecting both old first-generation and newer second-generation DAAs, regardless of their precise pharmacological mechanisms. However, those studies also reveal that although DAA-induced SVR reduces the risk of HCC occurrence, it does not completely eliminate it. This includes but it is not limited to patients with other concomitant risk factors, such as older age, male gender, and advanced fibrosis or established cirrhosis. Many possible explanations exist for this long-lasting risk, such as epigenetic alterations or oxidative stress, suggesting the persistence of hepatocyte damage and regeneration mechanisms. Some studies have also focused on the altered immunological profiles during and after DAA-induced HCV elimination, such as the persistence of regulatory T cells and the decrease of NKG2D after DAA treatments. Among other things, these changes may contribute to the reported possibility of early HCC occurrence [189].

Therefore, since SVR achievement does not eliminate the risk of HCC, current clinical guidelines still recommend surveillance for each individual, at least in principle. However, understandably, this strategy might not be feasible in a universal setting, despite the proven benefits. In this context, a more specific and cost-efficient surveillance system is needed to stratify patients based on their aforementioned individual risk factors. This is all the more true considering that the vast majority of HCC cases are generally observed within 12–24 months after viral response [255]. Thus, despite any residual controversy regarding whether DAAs could increase the risk of de novo HCC occurrence after achieving SVR, there is an urgent need for a risk stratification strategy in decision-making algorithms. This should combine not only the baseline characteristics of the patients and the most relevant pre-existing HCC risk factors, but also a selection of longitudinal predictors before and after DAA treatment, such as newly validated serum biomarkers [256].

## 7. Conclusions

Current evidence clearly demonstrates that the treatment of HCV-positive chronic hepatitis with DAAs is effective and safe, and that the risk of HCC should not be a concern or a reason to postpone or even deny any such curative regimens.

## Figures and Tables

**Table 1:** HCC pathogenesis in chronic HCV infection: direct HCV oncogenic mechanisms: (**A**) alterations in the regulation of cell proliferation; (**B**) alterations in cellular neoangiogenesis; (**C**) dysregulated gene expression patterns caused by alterations in cellular epigenetic processes.

Pathway/Factor/Gene Implicated	Mechanism(s)
(**A**)	
TERT pathway [94]	Promotion of HCC carcinogenesis and induction of more aggressive and undifferentiated HCC phenotypes
p53-p21-Rb pathway [95]	Induction of genomic instability leading to mitosis disorders and cell-cycle progression alterations
Wnt/β-catenin/c-Myc pathway [96]	Activation of a complex downstream cascade at multiple steps implicated in HCC stemness, progression, metastasis, and drug resistance
EGF receptor-mediated pathway [97]	Induction of a pro-inflammatory and pro-angiogenic signature which can promote HCC pathogenesis and emergence of more aggressive types
PI3K/Akt/mTOR pathway [98]	Promotion of cell proliferation and resistance to apoptosis in response to various harms like hypoxia and nutrient deficiency
(**B**)	
VEGF-A, angiopoietin-2, and PDGF [99]	Promotion of tumor angiogenesis
(**C**)	
DCAMKL1, Lgr5, CD133, AFP, CK19, LIN28A, c-Myc, and Nanog [100,101]	Induction of persistent self-renewal, sustained proliferation, tumor initiation, rarity within tumor tissue, expression of stem cell markers, differentiation into multiple lineages
CDKN2A [102]	Overcoming of stress-induced hepatocyte senescence
Notch [103]	Promotion of liver tumor formation, proliferation, invasion, and metastasis
Hedgehog [104]	Induction of HCC development, progression, and invasiveness
Rb [105]	Inhibition of apoptosis and promotion of chromosomal instability
HATs and HDACs [106]	Disturbance of double-strand break repair and promotion of HCC tumorigenesis
HNF4A and miR-122 [107]	Induction of hepatocyte proliferation and inflammation with promotion of HCC carcinogenesis and metastasis

Abbreviations: α-fetoprotein (AFP); protein kinase B (Akt); prominin-1 (CD133); cyclin-dependent kinase inhibitor 2A (CDKN2A); cytokeratin-19 (CK19); MYC proto-oncogene, bHLH transcription factor (c-Myc); doublecortin-like and CAM kinase-like 1 (DCAMKL1); epidermal growth factor (EGF); histone acetyltransferases (HATs); hepatocellular carcinoma (HCC); hepatitis C virus (HCV); histone deacetylases (HDACs); hepatocyte nuclear factor-4-alpha (HNF4A); leucine-rich repeat-containing G-protein coupled receptor 5 (Lgr5); lin-28 homolog A (LIN28A); mouse double minute 2 (MDM2); microRNA-122 (miR-122); mammalian target of rapamycin (mTOR); homeobox transcription factor Nanog (Nanog); neurogenic locus notch homolog protein (Notch); platelet-derived growth factor (PDGF); cyclin-dependent kinase inhibitor 1A (p21); tumor protein P53 (p53); retinoblastoma protein (Rb); telomerase reverse transcriptase (TERT); vascular-endothelial growth factor (VEGF); wingless-related integration site (Wnt).

**Table 2:** Other mechanisms of HCC pathogenesis in chronic HCV infection: (**A**) indirect host-related inflammatory response mechanisms; (**B**) bystander oncogenic mechanisms (concomitant liver and non-liver comorbidities associated with inflammation and fibrosis/cirrhosis).

Pathway/Factor Implicated	Mechanism(s)
(**A**)	
Innate immune system dysregulation [109]	Deactivation of the pro-inflammatory function of Kupffer cells
NK-cell-altered responses [109]	Induction of detrimental phenotypic and functional changes influencing the balance versus Th2 responses
CD8 Tcell-altered responses [110]	Contribution to the lack of resolution of HCV infection, which in turn is related to hepatic fibrogenesis
B-cell-altered responses [111]	Dismantling of possible ADCC of HCV-infected cells
(**B**)	
Metabolic disorders ^1^ [112,113,114,115]	Promotion of oxidative stress; increased expression of several signaling molecules known to be important in liver carcinogenesis
Co-infections ^2^ [116,117,118,119]	Promotion of inflammatory reactions and oxidative stress with induction of accelerated liver damage/fibrosis; possible carcinogenic synergy
Toxic ingestion ^3^ [120,121]	Promotion of oxidative stress, direct mutagenesis, aberrant methylation of DNA or protein on hepatocytes, immune system dysregulation

Abbreviations: antibody-dependent cellular cytotoxicity (ADCC); cluster of differentiation (CD); hepatitis B virus (HBV); hepatocellular carcinoma (HCC); hepatitis C virus (HCV); hepatitis delta virus (HDV); human immunodeficiency virus (HIV); natural killer cell (NK); T helper (Th); ^1^ obesity, diabetes mellitus, iron overload; ^2^ HBV, HDV, HIV, toxoplasma gondii; ^3^ inadequate alcohol consumption, aflatoxin B1 exposure.

**Table 3:** HCC pathogenesis in patients with previous chronic active hepatitis C who achieved SVR after treatment with DAAs: (**A**) alterations in immunosurveillance; (**B**) alterations in epigenetic regulations.

Pathway/Factor/Gene Implicated	Description	Main Physiopathological Mechanisms
(**A**)		
Reduction in type I IFNs production [182,183,184]	Inhibition of STAT1 phosphorylation Inhibition of immunosurveillance against viruses Inhibition of immunosurveillance against bacteria Inhibition of immunosurveillance against tumors	Increased expression of viral oncogenes Decreased expression of onco-suppressor genes Alterations of host and viral cell-cycle progression Reduced physiological apoptosis Mitochondrial dysfunction Increased genomic instability Reshaping of tissue microenvironment Altered cellular senescence
Increase in inflammation [185,186,187]	Increased VEGF levels Increased angiopoietin-2 levels	Altered angiogenesis, including tumor angiogenesis Enhanced cellular proliferation of numerous non-endothelial cells, including tumor cells
Reduction in anti-inflammatory responses [185,186]	Decreased IL-10 levels Decreased TNF-α levels	Dysregulation of the immune system Stimulation of cancer cell growth, proliferation, invasion, and metastasis Stimulation of tumor angiogenesis
Functional inhibition of NK cells [188,189]	Decreased NKG2D expression	Altered cellular stress sensing and response Strong inhibition of immune system activity Early termination of the immune response Blockage of immune checkpoint proteins
(**B**)		
Histone H3 [190,191,192,193]	H3K27ac H3K9ac H3K4Me3 H3K9Me3	High expression of SPHK1 oncogene Increased production of transcription factor Sp1 Cell apoptosis blocking and increased proliferation Increased tumor size Accelerated tumor progression High expression of SOX9 transcription factor Enhanced tumorigenesis Accelerated tumor progression Worse patient prognosis
		Persistent altered gene expression in hepatocytes (e.g., WNT10A, JUNB, FOLS2, MYCN, TNFAIP3, KLF4, EDN1) Dysregulation of host signaling pathways implicated in HCV and HCC proliferation Increased HCC development Increased cancer invasion and metastasis
Overexpression of DNMT1 [194,195,196,197] Hypermethylation of DKK3 with functional inhibition [194]	Aberrant activation of Wnt/β-catenin pathway EMT induction	Induction of HCC survival, proliferation, invasion, and neoangiogenesis

Abbreviations: direct-acting antivirals (DAAs); dickkopf WNT signaling pathway inhibitor 3 (DKK3); DNA methyltransferase 1 (DNMT1); epithelial–mesenchymal transition (EMT); endothelin 1 (EDN1); FOS-like 2 (FOLS2); hepatocellular carcinoma (HCC); trimethylation at the 4th lysine residue of the protein histone H3 (H3K4me3); acetylation at the 9th lysine residue of the protein histone H3 (H3K9ac); trimethylation at the 9th lysine residue of the protein histone H3 (H3K9Me3); acetylation of the lysine residue at N-terminal position of protein histone H3 (H3K27ac); interferon (IFN); interleukin (IL); JunB proto-oncogene, AP-1 transcription factor subunit (JUNB); KLF transcription factor 4 (KLF4); MYCN proto-oncogene, bHLH transcription factor (MYCN); natural killer (NK); natural killer group 2 member D (NKG2D); sex-determining region Y-box transcription factor 9 (SOX9); specificity protein 1 (Sp1); sphingosine kinase 1 (SPHK1); signal transducer and activator of transcription 1 (STAT1); tumor necrosis factor (TNF); TNF alpha-induced protein 3 (TNFAIP3); vascular endothelial growth factor (VEGF); wingless-related integration site (Wnt); Wnt family member 10A (WNT10A).

## Data Availability

Not applicable.

## Supplementary Materials

The following supporting information can be downloaded at https://www.mdpi.com/article/10.3390/v16121899/s1. Supplementary File S1: Clinical overview of HCV infection; Supplementary File S2: Data on occurrence of HCC after SVR achieved with (PEG)-interferon based regimens; Supplementary File S3: Occurrence of HCC: comparison between treatments with DAAs and IFN; Supplementary Table S1: Search terms and keywords used in the literature searches; Supplementary Table S2: Main current clinical recommendations about HCC management and surveillance before and after DAA treatment for HCV hepatitis in patients without active HCC when starting antiviral therapy; Supplementary Table S3: Main current clinical recommendations about HCC management and surveillance before and after DAA treatment in patients with active HCC when starting antiviral therapy; Supplementary Table S4: HCC risk calculators.
