# Supplementary material for: Chronic Hepatitis C Infection Treated with Direct-Acting Antiviral Agents and Occurrence/Recurrence of Hepatocellular Carcinoma: Does It Still Matter?
Source: Viruses. 2024 Dec 10;16(12):1899. doi: 10.3390/v16121899 (PMC11680226; doi:10.3390/v16121899)
Supplement: Supplementary file 1 [file viruses-16-01899-s001.zip › Supplementary File S1.pdf]

Review

Chronic hepatitis C infection treated with direct-acting antiviral agents and occurrence/recurrence of hepatocellular carcinoma: does it still matter?

Carlo Smirne, Maria Grazia Crobu, Irene Landi, Nicole Vercellino, Daria Apostolo, David James Pinato, Federica Vincenzi, Rosalba Minisini, Stelvio Tonello, Davide D'Onghia, Antonio Ottobrelli, Silvia Martini, Christian Bracco, Luigi Maria Fenoglio, Mauro Campanini, Alessandro Maria Berton, Alessia Ciancio and Mario Pirisi[2]

Supplementary File S1. Clinical overview of HCV infection

Although hepatitis C virus (HCV) incidence has generally declined in most countries in the last twenty years compared with the second half of the 20th century, substantial regional variation still exists. Notably, higher incidences are reported in Southern Europe (61.8 per 100,000) and in the Eastern Mediterranean (62.5 per 100,000). Country-specific data demonstrate that HCV incidence peaked in most countries between 1970 and 2005 [1].

As a recognized blood-borne pathogen, HCV continues to spread in many developing countries due to unsafe healthcare practices including the use of unsterile injections. Transmission outbreaks in dialysis units still occur even in high-income countries, so that screening is recommended for these patients [2]. Three key at-risk populations for HCV transmission are people who inject drugs (PWID), subjects with human immunodeficiency virus (HIV), and men who have sex with men (MSM) [3].

Regardless of the transmission route, HCV predominantly infects the liver. Most people infected with HCV do not spontaneously clear the infection, resulting in a life-long chronic illness. This persistent hepatic inflammation may cause acute and chronic necroinflammatory damage, leading to advanced liver diseases like fibrosis, cirrhosis, hepatocellular carcinoma (HCC) and, ultimately, death. In addition, HCV has been associated with several extrahepatic manifestations [4]. Unlike other infectious diseases, deaths from HCV have been increasing over the last 15 years, reaching an estimated 400,000 annually worldwide, merely mentioning liver-related deaths as more easily attributable [5]. However, only 20-30% of HCV mono-infected patients develop cirrhosis over their lifetimes, generally 30 years after acquiring the infection. Instead, HCV/HIV or HCV/hepatitis B virus (HBV) coinfections are notoriously associated with faster fibrosis progression to cirrhosis [6,7]. In any case, once achieved, cirrhosis represents the main risk factor for HCC (incidence rate: 1-4% per year) [8].

The natural history of HCV infection is even more multifaceted. Specifically, the complex interplay between host and virus in the acute infection phase results in either viral persistence (75-85%) or spontaneous clearance (15-25%) [7]. Host-related factors historically associated with spontaneous clearance of HCV include female sex, younger age, white ethnicity, symptomatic acute hepatitis, absence of the aforementioned coinfections, interferon lambda (IFN-λ) 3 and/or 4 genotypes, given HLA class II alleles (such as DQB1*02, DQB1*03, DRB1*04 and DRB1*11), HCV-specific T cell responses, and interleukin (IL)-28B genetic variations. However, spontaneous clearance of HCV depends also on factors directly related to the virus (like HCV genotype 1, high viral load, reduced diversity of HCV) [26,27].

Current guidelines recommend treatment for all patients with chronic hepatitis C, except those with a very short life expectancy not likely to improve after obtaining viral eradication and/or performing liver transplantation or other supportive treatments [28]. The purpose of therapy, whatever it may be, is to permanently eliminate HCV RNA, i.e. to achieve a sustained virologic response (SVR), which is usually associated with

normalizing of aminotransferase levels and halting of fibrotic progression. Treatment outcomes tend, in general, to be more favorable in patients with less fibrosis than in those with established cirrhosis.

For many years, interferon (IFN)-α or pegylated (PEG)-IFN-α combined with ribavirin (RBV) served as the standard-of-care antiviral therapy for chronic hepatitis C. However, these treatments eliminated the virus in just over half of the patients, with even lower success rates in more advanced fibrosis. Amongst other factors, the low response to these regimes was partially associated with specific IL-28B genotypes [29,30].

Nowadays, IFN-based regimens are no longer in use, due to the approval of IFN-free direct-acting antivirals (DAAs). These drugs target specific nonstructural (NS) viral proteins, thereby affecting multiple steps in the HCV replication life cycle [31]. Moreover, DAAs —only used in specific combinations in order to maximize effectiveness and minimize the development of resistance— are highly safe (unlike IFN-based therapies) and require shorter treatment durations [32]. As a result, virtually all patients are now eligible for these therapies, including those previously IFN-intolerant or ineligible [33–36], as well as individuals with advanced liver disease [37–45] and/or important comorbidities [46–56]. Some of these regimens are also approved for people with decompensated liver disease [37,39,57–59]. In such cases, treatment should be carried out in agreement with hepatologists. In any event, regimens containing protease inhibitors —which should always be handled with caution since some patients with cirrhosis have no evidence of inflammation based on aminotransferase levels— should not be used in decompensated cirrhosis [31,60].

From a broader perspective, while many first-generation DAAs required a selection based on HCV genotype, most currently available second-generation regimens are pangenotypic [7,61,62]. Overall, these drugs successfully eradicate HCV infection in more than 95% of cases, and are exceptionally well tolerated. As a result, within just a few years, and for the first time since the IFN era, the number of patients achieving SVR has steadily increased. This progress makes it, at least theoretically, conceivable that HCV infection could be eliminated in the next few years, provided that health authorities implement active screening and prompt treatment strategies. The rationale for this approach stems from the extensive evidence of the clinical benefits of achieving SVR. These mainly include a decrease in liver-related adverse events (such as hepatic decompensation and —although somewhat controversial, as it will be better clarified later on— HCC occurrence) and in non-liver-related complications (such as the development of insulin resistance and lymphomas). Additionally, SVR leads to improvements in life quality for patients, and long-term economic benefit for healthcare providers [50,63–66].
