# Supplementary material for: Chronic Hepatitis C Infection Treated with Direct-Acting Antiviral Agents and Occurrence/Recurrence of Hepatocellular Carcinoma: Does It Still Matter?
Source: Viruses. 2024 Dec 10;16(12):1899. doi: 10.3390/v16121899 (PMC11680226; doi:10.3390/v16121899)
Supplement: Supplementary file 1 [file viruses-16-01899-s001.zip › Supplementary File S2.pdf]

Review

Chronic hepatitis C infection treated with direct-acting antiviral agents and occurrence/recurrence of hepatocellular carcinoma: does it still matter?

Carlo Smirne, Maria Grazia Crobu, Irene Landi, Nicole Vercellino, Daria Apostolo, David James Pinato, Federica Vincenzi, Rosalba Minisini, Stelvio Tonello, Davide D'Onghia, Antonio Ottobrelli, Silvia Martini, Christian Bracco, Luigi Maria Fenoglio, Mauro Campanini, Alessandro Maria Berton, Alessia Ciancio and Mario Pirisi[2]

Supplementary File S2. Data on occurrence of HCC after SVR achieved with (PEG)-interferon based regimens

The role of interferon (IFN)-based therapies (standard IFN-α or pegylated (PEG)-IFN-α in combination with ribavirin) in reducing the risk of hepatocellular carcinoma (HCC) has been extensively studied, with findings highlighting a direct correlation with the baseline stage of hepatic fibrosis. This was true for both untreated and treated subjects: on average, 0.4% vs. 0.1% vs. 0.1% for stage ≤ F1, 2.0% vs. 0.8% vs. 0.1% for stage F2, 5.3% vs. 2.2% vs. 1.3% for stage F3, and 7.9% vs. 5.3% vs. 0.5% for stage F4, in untreated, non-sustained virologic response (SVR) and SVR patients, respectively [126]. These data were confirmed by numerous studies, as will be further detailed below. As a result, the following evidence on IFN-based regimens can now be considered as fully established:

- patients achieving SVR presented a significantly lower risk of HCC than those who did not [127,128];
- cirrhotic subjects at baseline had a greater post-SVR incidence of HCC compared to non-cirrhotic ones. It is quite difficult to make an exact estimate of such risk, but the annual rates reported in the literature generally were 2.7-4.5% vs. 0.1–2.8%, respectively, depending on additional risk factors such as age ≥ 50 years, male sex, non-white race and diabetes mellitus (DM) [127–131];
- even patients treated without achieving SVR appeared to have a slightly reduced HCC risk compared to untreated individuals [132,133], though this observation remains controversial and not fully explained [134,135];
- achieving SVR was associated to a significant decreased risk of all-cause (hazard ratio (HR): 0.26) and liver-related mortality (HR: 0.09), including the proportion attributable to the development of HCC. It also reduced the need for liver transplantation and re-transplantation (HR: 0.06). Incidentally, post-transplant survival markedly improved in the direct-acting antivirals (DAAs) era, for both patients with decompensated cirrhosis or HCC. Beyond liver-related causes of death, the biggest reduction after SVR was observed for cardiovascular-related ones, regardless of presence of cirrhosis (HR: 0.07) [21,63,69,132,136–143].

As mentioned before, the observation that cancer occurrence also decreased in the subjects who did not achieve SVR is indeed quite hard to explain. The most plausible interpretation could simply reside in the heterogeneity (e.g. in study design, patient inclusion criteria, cirrhosis prevalence, duration of administered treatments, diagnostic criteria for HCC) among clinical trials assessing the effectiveness of IFN-based therapies, resulting in some of the most common possible methodological errors [133,144]. However, there is a large body of evidence, mainly from Japan, suggesting that long-term IFN-treatments may slow fibrosis progression and reduce HCC development even without achieving SVR [126,145–147]. This suggests that IFN can reduce HCC risk not only via its obvious antiviral actions, but also through other direct and indirect anti-tumor effects, such as reducing hepatic inflammation, up-regulating the function of tumor suppressor genes, or directly inhibiting tumor cells [148–150]. In any case, the exact mechanism or mechanisms remain

uncertain. It is possible that IFN may delay tumor onset rather than actually preventing it, especially in cases of persistent HCV viremia or pre-existing cirrhosis.

In summary, while IFN-based treatments can lower the risk of HCC, they do not eliminate it entirely. Indeed, the risk remains elevated for years after achieving SVR, particularly in patients with cirrhosis and/or other aforementioned non-viral additional risk factors. For all these reasons, guidelines have long suggested initiating early HCV IFN-treatments, before significant liver fibrosis develops, and continuing HCC surveillance even after virological cure, especially for already cirrhotic subjects [130,151,152].
