# Supplementary material for: Chronic Hepatitis C Infection Treated with Direct-Acting Antiviral Agents and Occurrence/Recurrence of Hepatocellular Carcinoma: Does It Still Matter?
Source: Viruses. 2024 Dec 10;16(12):1899. doi: 10.3390/v16121899 (PMC11680226; doi:10.3390/v16121899)
Supplement: Supplementary file 1 [file viruses-16-01899-s001.zip › Supplementary File S3.pdf]

Review

Chronic hepatitis C infection treated with direct-acting antiviral agents and occurrence/recurrence of hepatocellular carcinoma: does it still matter?

Carlo Smirne, Maria Grazia Crobu, Irene Landi, Nicole Vercellino, Daria Apostolo, David James Pinato, Federica Vincenzi, Rosalba Minisini, Stelvio Tonello, Davide D'Onghia, Antonio Ottobrelli, Silvia Martini, Christian Bracco, Luigi Maria Fenoglio, Mauro Campanini, Alessandro Maria Berton, Alessia Ciancio and Mario Pirisi[2]

Supplementary File S3. Occurrence of HCC: comparison between treatments with DAAs and IFN

As previously mentioned, some authors initially reported significant higher rates of de novo or recurrent hepatocellular carcinoma (HCC) after direct-acting antivirals (DAAs) compared to interferon (IFN)-based regimes. However, this additional risk was not confirmed by other studies. On the contrary, most of the subsequent and cur-rent research has demonstrated that the risk of HCC occurrence is remarkably similar be-tween the two regimens [2].

The putative lower rate of HCC occurrence in those who obtained sustained virologic response (SVR) with IFN regimens could at least partially be accounted for the different baseline patient characteristics. Considering the significant heterogeneity of the studied populations, those who could tolerate IFN therapy were generally a very well-compensated group with minimal or moderate liver disease. Conversely, thanks to their minimal side effects, DAAs were proposed to patients with pre-existing risk factors for HCC development. These included older age (15 years on average), advanced fibrosis and defective hepatic function (with more frequent Child-Pugh B/C stages among persons with cirrhosis), severe comorbidities (including diabetes mellitus), and hepatitis C virus (HCV) genotype 1 infections. Some studies also suggested higher prevalence of previous HCC history in DAAs-treated subjects. Difficulties in data interpretation could include potential misclassifications of HCC before DAAs treatments, lack of an appropriate control group, generally shorter follow-up times in DAAs regimens and, sometimes, different methodologies of follow-ups. These imbalances could ultimately have led to a selection bias, explaining, at least, the higher numerical incidence of de novo HCC among the subjects exposed to the newer antivirals [125,151,176–178].

Finally, direct comparative studies between DAAs and IFN-based regimens are not easily feasible for heterogenicity of the compared populations [123]. However, the former ones seem as effective as the latter in reducing the incidence of HCC. More recent me-ta-analyses even demonstrated a possible protective effect of DAAs therapies. Overall, the median incidence rate of de novo HCC was 2.01/100 py (95% CI: 1.38-2.67) in the DAAs group and 1.45/100py (95% CI: 0.98-1.94) in the IFN-treated group. Additionally, HCC recurrence occurred in 16.76/100py (95% CI: 10.75-22.91) in the DAAs-treated group vs 20.04/100py (95% CI: 2.58-45.21) after IFN. Remarkably, after adjusting for age and cirrhosis, the HR was 0.58 (95% CI: 0.20-1.07) for HCC occurrence and 0.59 (95% CI: 0.24-1.03) for HCC recurrence after DAAs compared to IFN-based treatments [151,179,180].

Pending further final confirmation for what concerns this last issue, up to now it can however be stated with a good degree of confidence that, taken together, most of the evidence supports a substantial absence of difference between IFNs and DAAs in the residual risk of HCC (despite it was observed a trend towards a significantly lower chance of liver cancer for DAAs-treated versus untreated subjects). This argues against a specific mechanism of DAAs in promoting HCC, and suggests, in our opinion, that the debate regarding the impact of DAAs on HCC risk should draw to an end [2,125,181].
