# Supplementary material for: Chronic Hepatitis C Infection Treated with Direct-Acting Antiviral Agents and Occurrence/Recurrence of Hepatocellular Carcinoma: Does It Still Matter?
Source: Viruses. 2024 Dec 10;16(12):1899. doi: 10.3390/v16121899 (PMC11680226; doi:10.3390/v16121899)
Supplement: Supplementary file 1 [file viruses-16-01899-s001.zip › Supplementary Table S1.pdf]

Review

Chronic hepatitis C infection treated with direct-acting antiviral agents and occurrence/recurrence of hepatocellular carcinoma: does it still matter?

Carlo Smirne, Maria Grazia Crobu, Irene Landi, Nicole Vercellino, Daria Apostolo, David James Pinato, Federica Vincenzi, Rosalba Minisini, Stelvio Tonello, Davide D'Onghia, Antonio Ottobrelli, Silvia Martini, Christian Bracco, Luigi Maria Fenoglio, Mauro Campanini, Alessandro Maria Berton, Alessia Ciancio and Mario Pirisi

Supplementary Table S1. Search terms and keywords used in the literature searches. Combinations of groups of keywords, strings and Boolean operators (AND/OR) were used in the search strategy to retrieve the relevant publications The 'AND' Boolean operator was used to connect keyword groups while the 'OR' Boolean operator was used to connect each keyword in the groups. All keywords were searched in the title, abstract and keywords sections in each database. (**a**) and (**b**) refer to the queries in Pubmed-MEDLINE databases. (**c**) and (**d**) refer to the queries in Cochrane Library database.

	First keyword	Second keyword	Third keyword	Fourth keyword	Fifth keyword	Sixth keyword	Seventh keyword
(**a**)	chronic hepatitis C[1] OR HCV[2] OR hepatitis C[2]	antiviral agents[1] OR antiviral treatment[2] OR sofosbuvir-velpatasvir drug combination[1] OR glecaprevir and pibrentasvir[2] OR elbasvir-grazoprevir[2] OR telaprevir[2]	sustained virological response[1] OR SVR[2]	hepatocellular carcinoma[1] OR HCC[2]	publication type[3]	y_10[4]	
(**b**)	chronic hepatitis C[1] OR HCV[2] OR hepatitis C[2]	antiviral agents[1] OR antiviral treatment[2] OR sofosbuvir-velpatasvir drug combination[1] OR glecaprevir and	sustained virological response[1] OR SVR[2]	hepatocellular carcinoma[1] OR HCC[2]	publication type[3]	y_10[4]	liver cirrhosis[1] OR cirrhosis[2]

		pibrentasvir[2] OR elbasvir-grazoprevir[2] OR telaprevir[2]					
(**c**)	chronic hepatitis C OR hepatitis C OR HCV	DAA OR antiviral agents OR antiviral treatment OR sofosbuvir OR sofosbuvir-velpatasvir OR glecaprevir OR pibrentasvir OR elbasvir OR grazoprevir	sustained virological response OR SVR	hepatocarcinoma OR HCC	publication type[5]	y_10[4]	
(**d**)	chronic hepatitis C OR hepatitis C OR HCV	DAA OR antiviral agents OR antiviral treatment OR sofosbuvir OR sofosbuvir-velpatasvir OR glecaprevir OR pibrentasvir OR elbasvir OR grazoprevir	sustained virological response OR SVR	hepatocarcinoma OR HCC	publication type[5]	y_10[4]	liver cirrhosis OR cirrhosis

Abbreviations: hepatitis C virus (HCV); hepatocellular carcinoma (HCC); sustained virologic response (SVR); y (years). [1] MeSH Terms. [2] All Fields. [3] The following filters were respectively applied to four different queries: all fields; clinical trial OR randomized controlled trial; review OR systematic review; meta-analysis. [4] Publication date filter. [5] The following filter was applied: Cochrane central register of controlled trials
