# Supplementary material for: Chronic Hepatitis C Infection Treated with Direct-Acting Antiviral Agents and Occurrence/Recurrence of Hepatocellular Carcinoma: Does It Still Matter?
Source: Viruses. 2024 Dec 10;16(12):1899. doi: 10.3390/v16121899 (PMC11680226; doi:10.3390/v16121899)
Supplement: Supplementary file 1 [file viruses-16-01899-s001.zip › Supplementary Table S2.pdf]

Review

Chronic hepatitis C infection treated with direct-acting antiviral agents and occurrence/recurrence of hepatocellular carcinoma: does it still matter?

Carlo Smirne, Maria Grazia Crobu, Irene Landi, Nicole Vercellino, Daria Apostolo, David James Pinato, Federica Vincenzi, Rosalba Minisini, Stelvio Tonello, Davide D'Onghia, Antonio Ottobrelli, Silvia Martini, Christian Bracco, Luigi Maria Fenoglio, Mauro Campanini, Alessandro Maria Berton, Alessia Ciancio and Mario Pirisi

Supplementary Table S2. Main current clinical recommendations about HCC management and surveillance before and after DAA treatment for HCV hepatitis in patients without active HCC when starting antiviral therapy.

HCC recommended surveillance before DAAs therapy initiation	DAAs therapy recommendations	Post-DAAs therapy estimated risk of *de novo* or recurrent HCC	Post SVR recommended HCC surveillance
F3-F4 patients: imaging with an abdominal US exam<br>F0→F2 patients: US exam [1]	Unless specific conditions/ contraindications are present, all patients should be started on DAAs as soon as possible, regardless of disease stage. Therapy is contraindicated only in patients with severe hepatic impairment [2]	DAAs treatment has been linked to a lower risk of incident HCC. The relative risk reduction in F0→F3 and F4 patients is comparable	F3-F4 patients: indefinite monitoring every 6 months, using US ± AFP testing. Shorter monitoring periods or the adoption of different surveillance modalities are not supported by current data
			F0→F2 patients: When indicated, US follow-up may be proposed every 12 months [3]

Abbreviations: α-fetoprotein (AFP); direct-acting antivirals (DAAs); no fibrosis (F0); mild fibrosis (F1); moderate fibrosis (F2); advanced liver fibrosis (F3); liver cirrhosis (F4); hepatocellular carcinoma (HCC); hepatitis C virus (HCV); sustained virologic response (SVR); ultrasound (US); : [1] the level of recommendation for such surveillance is less strong, and should be decided on an individual basis; [2] class C according to Child-Pugh score; [3] APASL recommends US ± AFP testing every 6 months for the first two years after SVR.
