# Supplementary material for: Chronic Hepatitis C Infection Treated with Direct-Acting Antiviral Agents and Occurrence/Recurrence of Hepatocellular Carcinoma: Does It Still Matter?
Source: Viruses. 2024 Dec 10;16(12):1899. doi: 10.3390/v16121899 (PMC11680226; doi:10.3390/v16121899)
Supplement: Supplementary file 1 [file viruses-16-01899-s001.zip › Supplementary Table S3.pdf]

Review

Chronic hepatitis C infection treated with direct-acting antiviral agents and occurrence/recurrence of hepatocellular carcinoma: does it still matter?

Carlo Smirne, Maria Grazia Crobu, Irene Landi, Nicole Vercellino, Daria Apostolo, David James Pinato, Federica Vincenzi, Rosalba Minisini, Stelvio Tonello, Davide D'Onghia, Antonio Ottobrelli, Silvia Martini, Christian Bracco, Luigi Maria Fenoglio, Mauro Campanini, Alessandro Maria Berton, Alessia Ciancio and Mario Pirisi

Supplementary Table S3. Main current clinical recommendations about HCC management and surveillance before and after DAA treatment in patients with active HCC when starting antiviral therapy. (**A**) Patients with very early or initial stage HCC; (**B**) Patients with intermediate or advanced stage HCC.

**Recommended management of HCC**	**DAAs therapy considerations/ recommendations**	**Post-DAAs therapy estimated risk of HCC after HCC complete response**	**Post SVR recommended HCC surveillance in patients with HCC complete response**
		(**A**)	
As per clinical indication (generally, LR, RFA or OLT)	Active HCC is associated with a slight, but still significant, decrease in SVR rates with DAAs	It is unknown whether DAAs therapy is associated with different *de novo* HCC risk, time to recurrence or aggressiveness of recurrent HCC	HCC surveillance with dynamic contrast-enhanced CT or MRI (instead of standard US) may be considered indefinitely every 3-6 months, if economic restraints are not a major issue. Current evidence does not support more frequent monitoring [1]
	Patients with HCC who are candidates for LR or RFA should postpone DAAs until their HCC treatment is concluded		
	In all patients who underwent curative HCC therapies, DAAs may be postponed for further 4-6 months, in order to confirm complete HCC response		

(**B**)			
As per clinical indication. Unless the patient is placed on the waiting OLT list, tumor treatment should always precede any antiviral treatment	Active HCC is associated with a slight but still significant decrease in SVR rates with DAAs therapies	Same as above	Same as above
In HCC candidates for OLT the timing of DAAs should take in consideration various factors [2]	There are insufficient data to fully evaluate the benefits and cost-effectiveness of DAAs therapies. Decisions in these patients should consider various factors [3]		

Abbreviations: α-fetoprotein (AFP); computed tomography (CT); direct-acting antivirals (DAAs); hepatocellular carcinoma (HCC); hepatitis C virus (HCV); liver resection (LR); magnetic resonance imaging (MRI); orthotopic liver transplantation (OLT); radiofrequency ablation (RFA); sustained virologic response (SVR); ultrasound (US). [1] APASL recommends US + AFP testing every 4 months, with the possibility of adding CT, MRI or contrast-enhanced US; [2] such as median wait times, availability of HCV-positive organs, and liver dysfunction degree: in any case, the current trend is increasingly to start treatments earlier even before OLT [3] such as tumor burden, extent of liver dysfunction, life expectancy, and also patient preferences.
