# Supplementary material for: Chronic Hepatitis C Infection Treated with Direct-Acting Antiviral Agents and Occurrence/Recurrence of Hepatocellular Carcinoma: Does It Still Matter?
Source: Viruses. 2024 Dec 10;16(12):1899. doi: 10.3390/v16121899 (PMC11680226; doi:10.3390/v16121899)
Supplement: Supplementary file 1 [file viruses-16-01899-s001.zip › Supplementary Table S4.pdf]

Review

Chronic hepatitis C infection treated with direct-acting antiviral agents and occurrence/recurrence of hepatocellular carcinoma: does it still matter?

Carlo Smirne, Maria Grazia Crobu, Irene Landi, Nicole Vercellino, Daria Apostolo, David James Pinato, Federica Vincenzi, Rosalba Minisini, Stelvio Tonello, Davide D'Onghia, Antonio Ottobrelli, Silvia Martini, Christian Bracco, Luigi Maria Fenoglio, Mauro Campanini, Alessandro Maria Berton, Alessia Ciancio and Mario Pirisi

Supplementary Table S4. HCC risk calculators.

Tool	Description
FIB-4 [1]	Age (years) × AST [U/L] / (platelet count [10^9/L] × (ALT [U/L])$^{1/2}$
aMAP [2]	((age [years] × 0.06 + gender × 0.89 (male: 1, female: 0) + 0.48 × (($\log_{10}$ bilirubin [μmol/L] + (albumin [g/L] × −0.085)) - 0.01 × platelet count [10^3/mm^3]) + 7.4) / 14.77 × 100
GALAD [2]	−10.08 + 1.67 × gender (male: 1, female: 0) + 0.09 × age + 0.04 × AFP-L3% [3] + 2.34 × $\log_{10}$AFP × $\log_{10}$DCP

Tool	Predictors	Predictor categories
VA HCC model [2,4]	AFP, ng/mL	≤ 4
		> 4 - 6.5
		> 6.5 - 10.5
		> 10.5 - 14.71
		> 14.71
	Sex	Male
		Female
	Age, years	≤ 56
		> 56 - 60
		> 60 - 64
		> 64 - 67
		> 67

	BMI, Kg/m^2	< 20
		20- 25
		25 - 30
		30- 35
		> 35
	Race/Ethnicity	White, non-Hispanic
		Black, non-Hispanic
		Hispanic
		Other
		Declined to answer, missing
	HCV Genotype	Non-3
		Genotype 3
	Hemoglobin, g/dL	> 15.7
		> 14.8 - 15.7
		> 13.7 - 14.8
		> 12.7 - 13.7
		≤ 12.7
	Platelet count, x 10^9/L	> 167
		> 123 - 167
		> 87 - 123
		> 61 - 87
		≤ 61
	Albumin, g/dL	> 4.0
		> 3.7 – 4.0
		> 3.3 - 3.7
		> 2.9 - 3.3
		≤ 2.9
	INR, Units	≤ 1.00
		> 1.00 - 1.20
		> 1.20 - 1.34
		> 1.34
	AST/√ALT	≤ 6.50
		6.50 - 8.49
		8.49 - 11.01
		11.01 - 13.90
		> 13.90
GES score [2]	Sex	Male
		Female
	Age, years	≤ 54

		> 54
	Fibrosis stage	F3<br>F4
	Albumin, g/dL	≥ 3.8<br>< 3.8
	AFP, ng/mL	≤ 20<br>> 20
ADRESS-HCC model [2]	Age, years	
	Diabetes	Present<br>Absent
	Race/Ethnicity	Non-Hispanic white<br>Non-white or Hispanic
	Etiology	Autoimmune<br>Alcohol/metabolic<br>Viral
	Sex	Male<br>Female
	Child-Pugh score (severity of liver disease)	5 → 15
Watanabe score [2]	Sex	Male<br>Female
	Diabetes mellitus	Present<br>Absent
	White blood cell count, n x μL	≥ 5,600<br>4,223–5,600<br>≤ 4,223
	Prothrombin activity, %	≥ 99.7<br>87.1–99.7<br>≤ 87.1
	FIB-4 index at EOT [5]	≤ 1.7<br>1.7–2.8<br>≥ 2.8
	FIB-4 index at SVR	≤ 1.84<br>1.84–2.90<br>≥ 2.90
	AFP at EOT, ng/mL	≤ 3.0<br>3.0–4.6<br>≥ 4.6
	AFP at SVR, ng/mL	≤ 2.8

		2.8–4.0<br>≥ 4.0
RNN HCC model [2]	Age [6], sex, race, HCV genotype [7]<br>Cirrhosis development, SVR achievement, BMI [8]<br>Bilirubin, AST, AST/ULN, ALT, ALT/ULN, AFP, AFP/ULN, ALP, ALP/ULN, albumin, AST/ALT, FIB-4 score, APRI, blood urea nitrogen, creatinine, glucose, INR, hemoglobin, white blood cell count, platelet count, sodium, potassium, chloride, total protein [8,9]	

Abbreviations: α-fetoprotein (AFP); α-fetoprotein L1 glycoform (AFP-L1); LCA-reactive α-fetoprotein L3 glycoform (AFP-L3); alkaline phosphatase (ALP); AST-platelet ratio index (APRI); aspartate transaminase (AST); alanine aminotransferase (ALT); body mass index (BMI); des-gamma-carboxy-prothrombin (DCP); advanced liver fibrosis (F3); liver cirrhosis (F4); fibrosis-4 (FIB-4); hepatocellular carcinoma (HCC); hepatitis C virus (HCV); international normalized ratio (INR); lens culinaris agglutinin (LCA); recurrent Neural Network (RNN); sustained virologic response (SVR); upper limit of normal (ULN); Veterans Affairs (VA). [1] simplified scoring system. [2] multivariable HCC risk calculator. [3] calculated as follows: AFP-L3% = AFP-L3 concentration / (AFP-L1 concentration + AFP-L3 concentration) × 100. [4] Four groups (cirrhosis/SVR, cirrhosis/no SVR, no cirrhosis/SVR no cirrhosis/no SVR. Available online: www.hccrisk.com (accessed on 23 September 2024). [5] at end of treatment (EOT). [6] at cirrhosis diagnosis. [7] all baseline predictors. [8] all longitudinal predictors. [9] all laboratory blood tests.
